# Reconstruction of necrotic submandibular salivary gland using mesenchymal stem cells

**DOI:** 10.1016/j.heliyon.2020.e05162

**Published:** 2020-10-08

**Authors:** Shamsoulmolouk Najafi, Haleh Nosrati, Zahra Faraji, Abdolreza Mohamadnia, Sadegh Shirian, Seyed Mostafa Mortazavi, Naghmeh Bahrami

**Affiliations:** a Dental Research Center, Tehran University of Medical Sciences, Tehran, Iran; b Dept. of Oral & Maxillofacial Medicine, School of Dentistry, Tehran University of Medical Sciences, Tehran, Iran; c Dept of Oral & Maxillofacial Medicine, School of Dentistry, International Campus, Tehran University of Medical Sciences, Tehran, Iran; d Tehran University of Medical Sciences, Tehran, Iran; e Tehran University of Medical Science, Tehran, Iran; f Chronic Respiratory Diseases Research Center, National Research Institute of Tuberculosis and Lung Diseases (NRITLD), ShahidBeheshti University of Medical Sciences, Tehran, Iran; g Department of Biotechnology, School of Advanced Technologies in Medicine, ShahidBeheshti University of Medical Sciences, Tehran, Iran; h Department of Pathology, School of Veterinary Medicine, Shahrekord University, Shahrekord, Iran; i Shiraz Molecular Pathology Research Cenetr, Dr Daneshbod Lab, Shiraz, Iran; j Shefa Neuroscience Reseach Center, Tehran, Iran; k Department of Oral and Maxillofacial Surgery, School of Dental Medicine, Alborz University of Medical Science, Karaj, Iran; l Department of Tissue Engineering and Applied Cell Sciences, School of Advanced Technologies in Medicine, Tehran University of Medical Sciences, Tehran, Iran; m Craniomaxillofacial Research Center, Tehran University of Medical Sciences, Tehran, Iran

**Keywords:** Biochemistry, Cancer research, Cell biology, Molecular biology, Regenerative medicine, Stem cell research, Dry mouth, Salivary glands reconstruction, Stem cell transplantation

## Abstract

**Background:**

The efficacy of mesnchymal stem cells (MSCs) to treat the necrotic tissue of salivary glands (SGs) has yet investigated.

**Objective:**

This study was conducted to investigate the potential capacity of MSCs to restore the function and regenerate the necrotic submandiular gland in the rat animal model.

**Methods:**

Twenty-one Sprague–Dawley rats were provided from a breeding colony and randomly divided into three groups including the positive control or induced SG atrophy without treatment, the treatment group or induced SG atrophy with MSCs isolated transplantation and the negative control group consists of healthy rats. The atrophic and necrotic submandiular gland was induced using intraoral duct ligation of the main duct of submandiular gland for one month. The isolated stem cells were confirmed using flow cytometry for CD90 and CD 105. The isolated MSCs were cultured and injected to submandiular gland and the potential efficacy of MSCs to treat the atrophic submandibular glands was evaluated using histopathology on two weeks post-transplantation. To detect the acinar cell protein secretory granules, Alcian Blue and periodic acid shift (PAS) staining were done. For the demonstration of mitotic index or proliferation rate of the SG epithelia tissue, Ki-67 and Smbg proteins expression were evaluated using immunohistochemistry.

**Results:**

The locally injected MSCs could regenerate the overall histological structure of the necrotic submandibular gland tissue within 2 weeks of post-transplantation. Alcian Blue and PAS staining indicated that the mean amount of serous and mucin secretions in the treatment group was significantly increased compared to the positive control groups. We have also found that the treatment group significantly express higher Ki-67 protein, as a diagnostic marker for cell mitosis and proliferation rate, and lower Smbg protein, as a diagnostic marker, for damage to the submandibular gland than that of control group.

**Conclusion:**

This study demonstrates the therapeutic benefits of MSCs isolated from the SG in treating atrophic and necrotic SGs in a rat model. MSCs may be potential candidates for cell-based therapies targeting hypofunction of SG induced by a range of diseases or because of surgery and radiotherapy of head and neck cancers.

## 1 Introduction

Salivary glands with various physiological functions are critical structures in the oral cavity. They are important for maintenance of the oral cavity homeostasis. The secreted saliva contains electrolytes and antibacterial compounds as well as several enzymes to protect the teeth and surrounding soft tissues. The SGs provide the lubrication of the oral cavity requiring for speech and perception of food taste (Porcheri and Mitsiadis, 2019; de Paula et al., 2017). Their normal daily production of saliva, average of 1–1.5 L, has multiple essential functions to the health and function of the oral cavity and gastrointestinal tract (Mattingly et al., 2015). Various diseases such as physical traumas, infections, autoimmune diseases, and cancer, can alter the SGs functionality, greatly impacting the patient's quality of life (Porcheri and Mitsiadis, 2019). Irreversible SGs dysfunctions and their associated symptoms are named Xerostomia. It is a subjective sensation of oral dryness and is a hallmark of several systemic diseases, such as granulomatous diseases, Sjögren's syndrome, cystic fibrosis, uncontrolled diabetes, graft versus host disease, human immunodeficiency virus infection, thyroid disease, and late stage liver disease (Lombaert et al., 2017). Xerostomia can be caused by surgical injury, radiotherapy of head-and-neck cancers, aging and genetic anomalies (Emmerson and Knox, 2018; Millsop et al., 2017). Despite multiple treatment options for SG hypofunction such as systemic parasympathomimetic, sialogogues, and artificial saliva substitutes their effect are challenging. Common treatment strategies for SG hypofunction are temporary and the SGs are not restored functionally (See et al., 2019). Stem cell-based therapy has been recently introduced for treatment of SGs hypofunction (Sui et al., 2020). The first SG tissue regeneration was done using transplanting autologous SG epithelial cells in rodents to increase salivary function (Lombaert et al., 2008). MSCs are multi-potential cells that can be isolated from varied organ tissues. They have potential ability to differentiate to heterogeneous populations of multipotent stromal cell (Nicolay et al., 2015). The potential of MSC to restore SG function in the induced-damage by irradiation has been demonstrated in animal model (Lim et al., 2013). The treatment of external radiation-induced SG damages using different types of stem cells has been widely studied (Choi et al., 2018; Nicolay et al., 2015; Lim et al., 2013). Bone regeneration by MSC transplantation in a rabbit model of avascular necrotic femoral head due to the upregulated expression of the chemokines stromal cell-derived factor-1 and monocyte chemoattractant protein-1 has been recently reported (Wu et al.,. 2019). However, the efficacy of MSC to treat the necrotic tissue of SGs has yet investigated. Since, the necrotic tissue is usually resected and eliminated from the organ; it seems this can be innovative method for treatment of severe SG damages. Therefore, this study was conducted to investigate the potential capacity of MSCs to restore the function and regenerate the necrotic SG in the rat animal model.

## 2 Material and method

Twenty-one Sprague–Dawley rats (200–220g) were provided from a breeding colony. They were fed with commercial chow and kept in a temperature-controlled room (22 ± 1 °C) with a 12 h light:12 h darkness cycle for 1 week before the beginning of experiment to adapt the new environment. The rats randomly divided into three groups including the positive control or induced SG atrophic/necrotic without treatment, the treatment group or induced SG atrophy/necrotic with MSCs transplantation and the negative control group consists of healthy rats. The present study was approved by the Medical Ethics Committee of the Research Vice Chancellor of Tehran University of Medical Sciences (4787.1396.REC.DENTISTRY.TUMS.IR) and was done in accordance with NIH guidelines to care and used of the laboratory animals.

### 2.1 Animal surgery and the creation of SG damaged models

**Figure 1:**
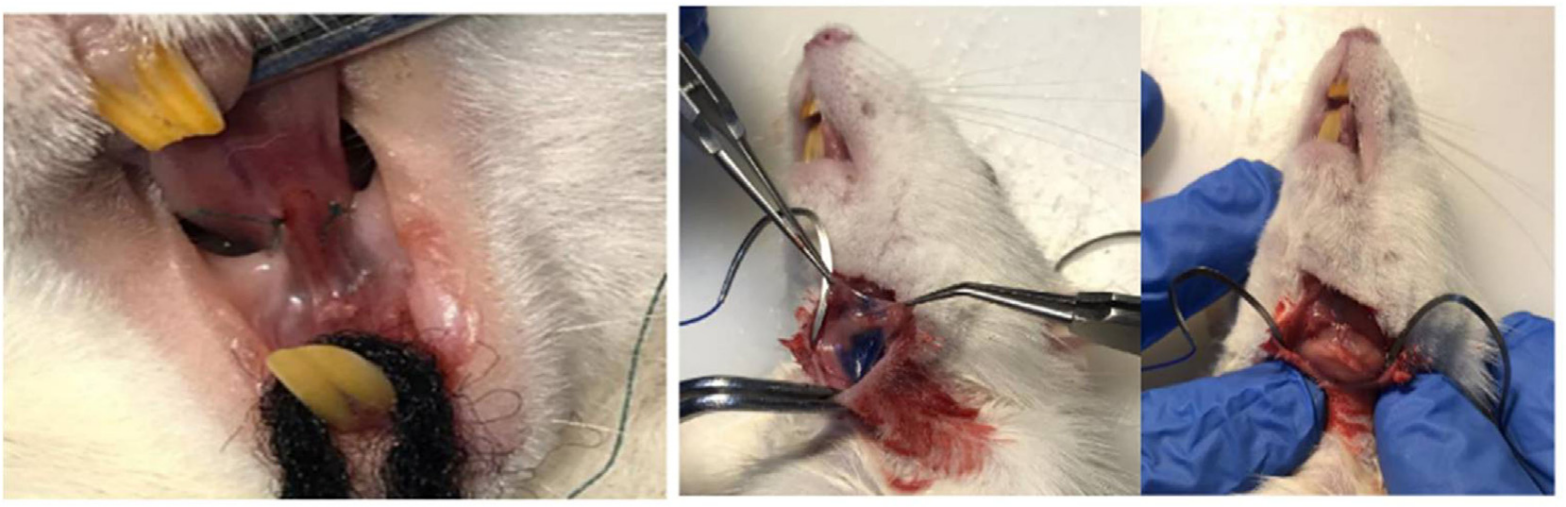
Salivary duct ligator and finding salivary glands and injecting stem cells with the blue color of Trypan Blue.

The animals were anesthetized with intra-peritoneal injection of ketamine-xylazine. In the sham and treatment groups the duct opening for the submandibular gland was found within the floor of the mouth and a narrow incision, five millimeters away from the duct along the gland, was made and sutured with an 8-0 thread (Figure 1). The salivary ducts were ligated for 1 month.

### 2.2 Isolation of MSC

MSCs were isolated from submandibular SG of healthy rats as previously described by Nanduri et al. (2011). The isolated MSCs were cultured in DMEM (Gibco, USA) media supplemented with 10%, 100 IU penicillin as well as 100IU streptomycin at 5% CO_2_ and 37 °C.

**Figure 2:**
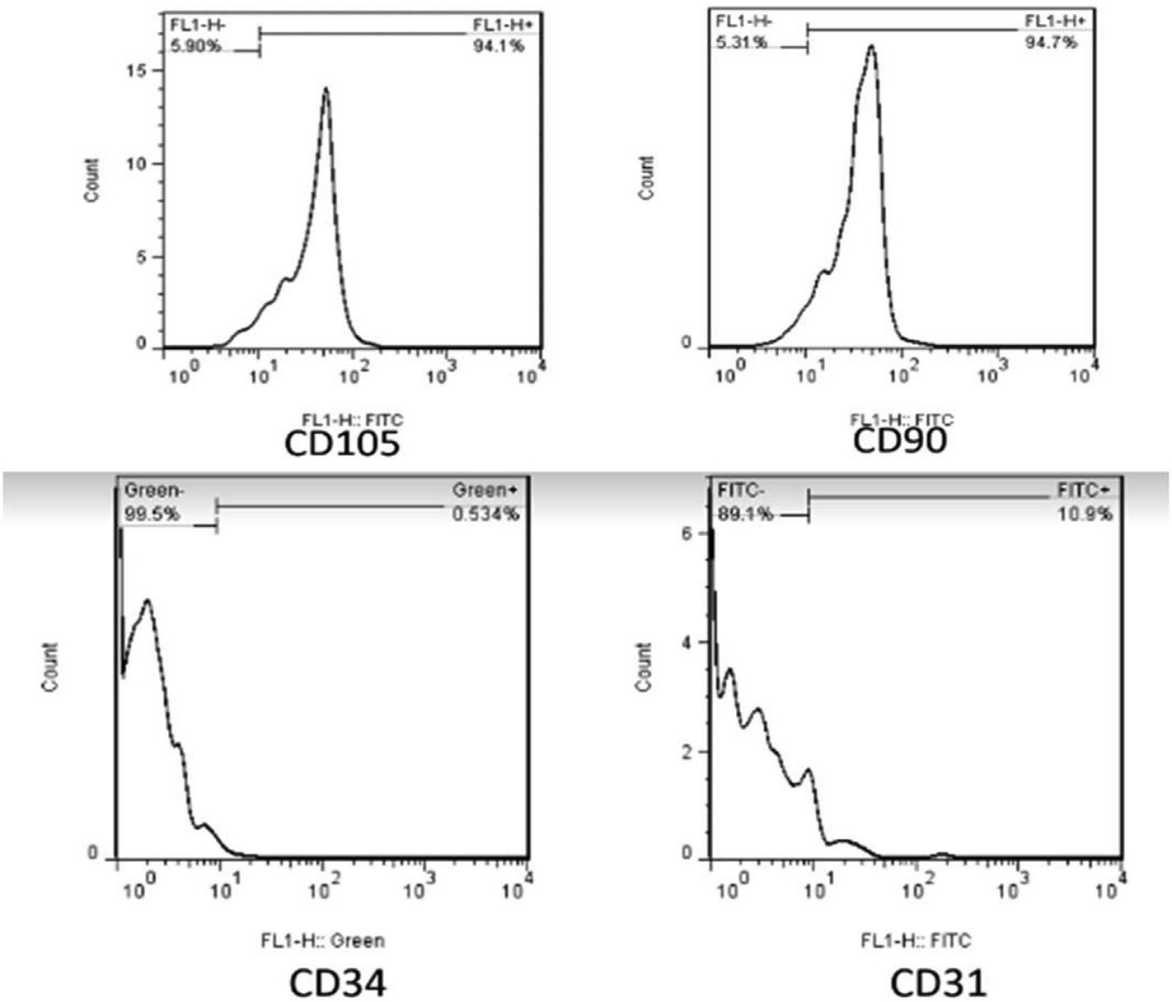
The expression of CD 105, CD90, CD31 and CD34 markers using flow cytometry.

The cells were characterizated by flow cytometry when reach to approximately 80% confluency using CD31, CD105, CD90, CD34, and HLA- DR markers. The passage 3 MSCs were used for the experiments after confirmation. MSCs, one million per kilogram of body weight, were transplanted into submandibular gland on one month post-induced SG damage (Figure 2).

### 2.3 Microscopic evaluation

The rats were scarified on two weeks post-transplantation and the submandibular glands were removed and fixed in 10% formalin buffer (Merck, Germany). The fixed tissues were embedded in paraffin, sectioned into 5μm and stained with hematoxylin and eosin staining as well as by Periodic Acid Schiff (PAS) and Alcian Blue staining.

### 2.4 Immunohistochemistry

Immunonostaining of the submandibular gland tissue was conducted for quantification of Ki67 and submandibular gland secretory protein b (Sbmg) proteins expression as previously described by Soleimanpuor et al. (2012). In brief, the paraffin sections, 3μm in thickness, were cut, deparaffinized and rehydrated. The samples were treated in 3% hydrogen peroxide for 5 min at room temperature to block endogenous peroxidase activity. The heat-induced antigen retrieval was done using 0.01 mol/L Tris buffer containing 0.001 mol/L EDTA at pH 9.0 for 20 min in a microwave oven. Subsequently, the samples were incubated with rabbit Anti-Ki67 and Sbmg primary antibodies (Abcam, USA; 1:200) for 1h at room temperature. The incubated samples with primary antibodies were washed in PBS two times each of 5 min. The Envision+ (DakoCytomation) detection system was used to detect the immunoreactivity and developed with diaminobenzidine (DakoCytomation).

### 2.5 Statistical analysis

Data were analyzed using SPSS software version 22. All data were expressed as mean ± standard deviation (SD). The mean difference was analyzed using the One Way ANOVA followed by post hoc Tukey. P < 0.05 was considered statistically significant.

## 3 Results

### 3.1 Flow cytometry

The nature of MSC was confirmed using the flow cytometry using highly expression of CD105 (94.1%), CD90 (94.7%) and extremely low expression of CD31 (10.9%), and CD34 (0.534%) markers.

### 3.2 Immunohistochemical findings

**Figure 3:**
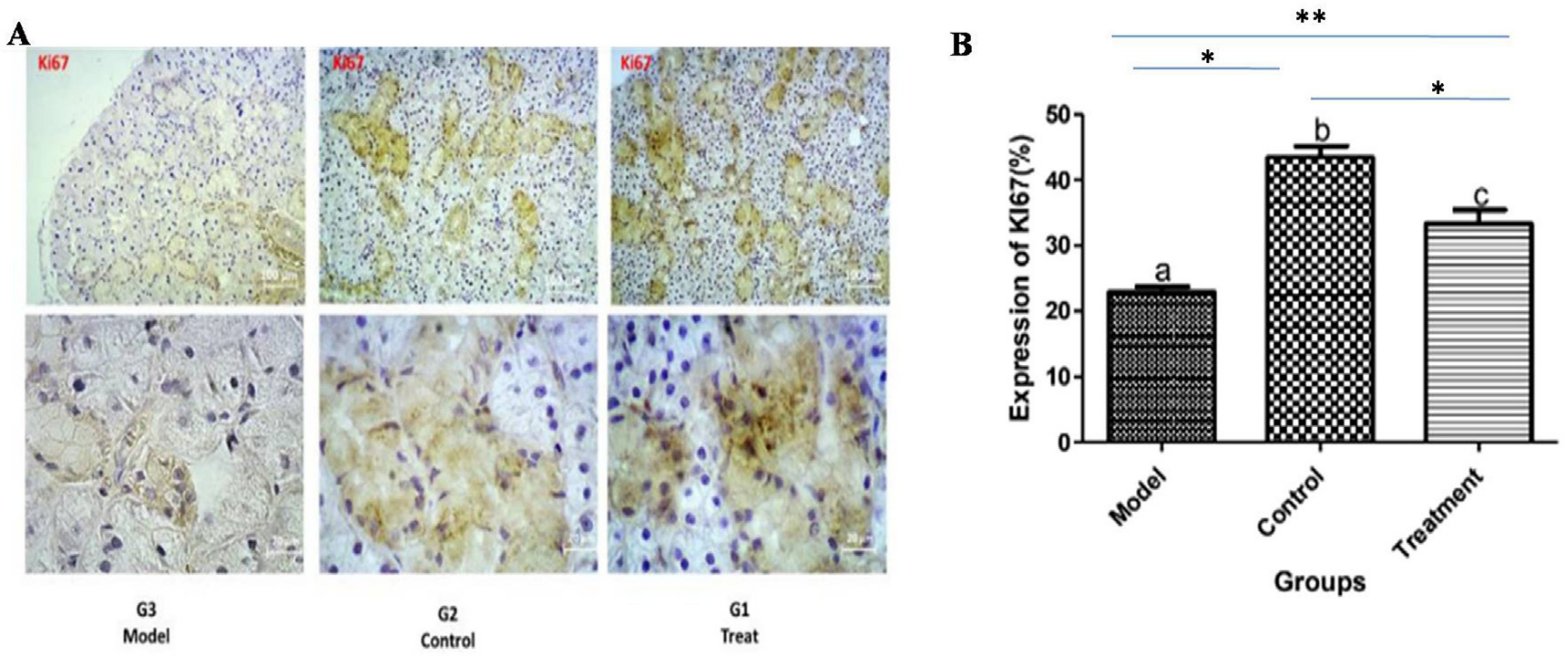
A: Immunoreactivity of Ki-67 in various groups, B: The mean expression of Ki-67 expression in all group. ∗∗indicates P < 0.001, ∗ indicates P < 0.01.

**Figure 4:**
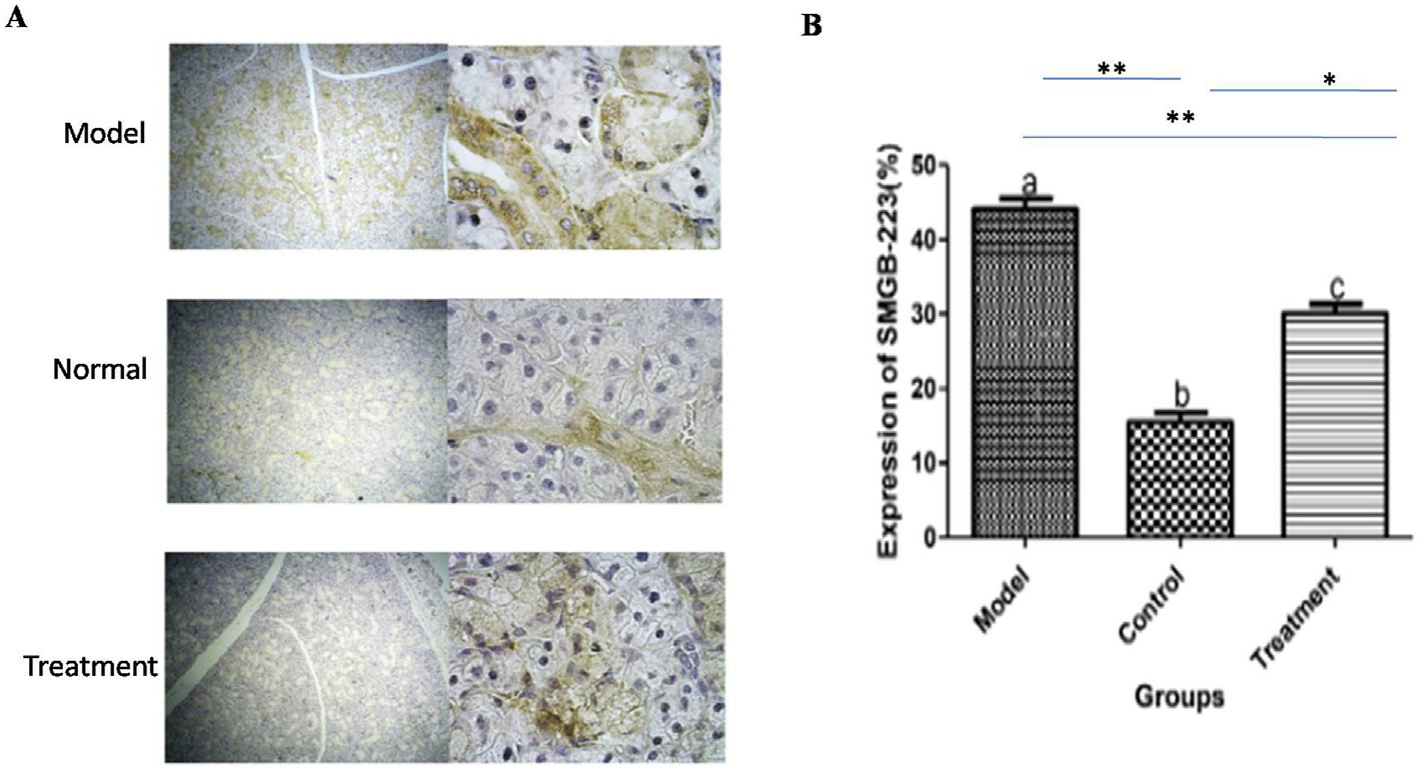
A: Immunoreactivity of Smbg in various groups, B: The mean expression of Smbg expression in all group. ∗∗indicates P < 0.001, ∗ indicates P < 0.01.

In the treatment group, the mean percentage expression of Ki-67 was significantly higher and lower than those of the positive and negative control groups, respectively (P < 0.001) (Figure 3). However, in the treatment group the mean percentage expression of Sbmg was significantly lower and higher than those of the positive and negative control groups, respectively (P < 0.001) (Figure 4).

### 3.3 PAS and Alcian blue staining findings

**Figure 5:**
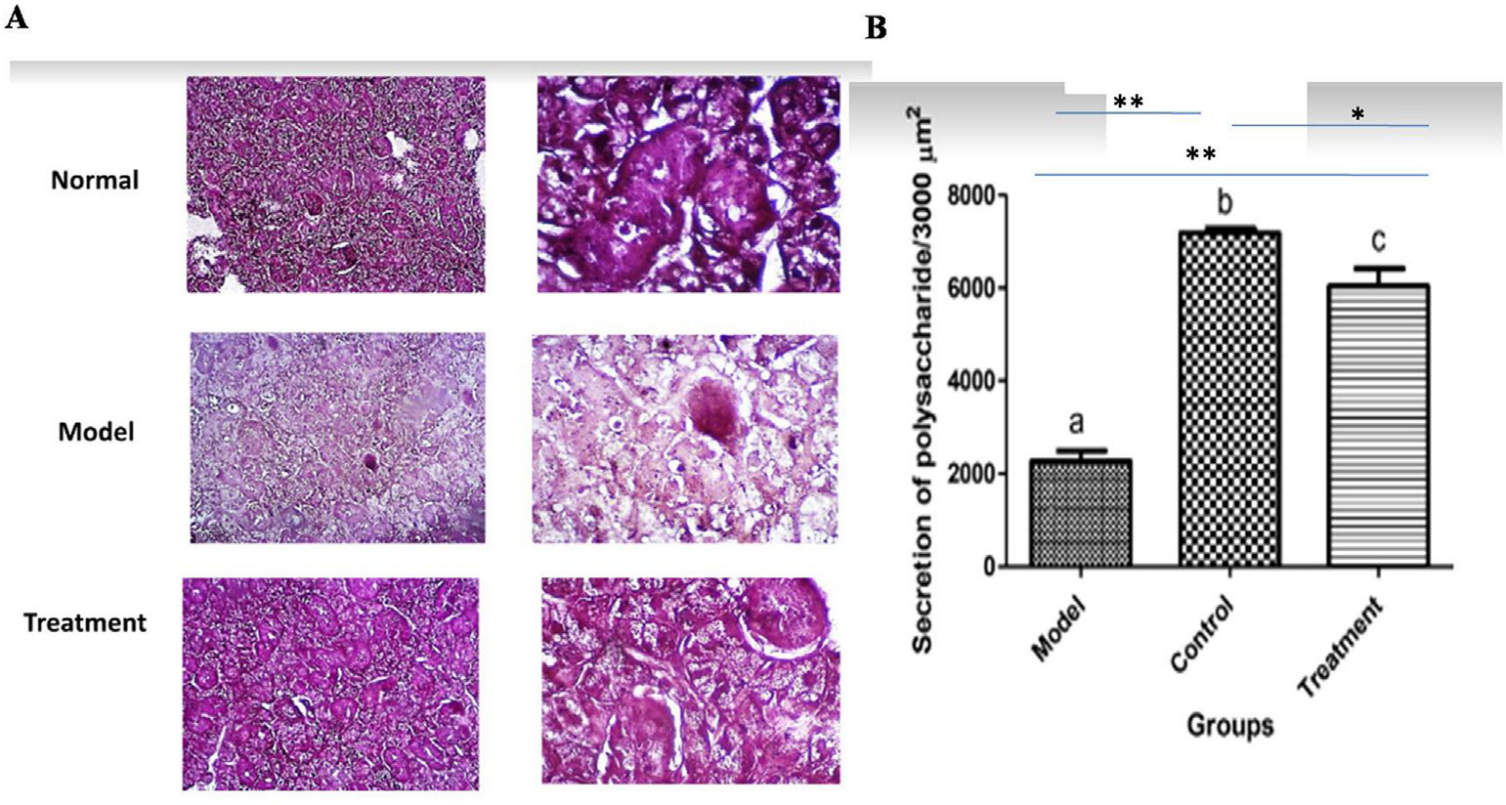
A: PAS staining in various groups, B: The mean secretion serous of in all group. ∗∗indicates P < 0.001, ∗ indicates P < 0.01.

**Figure 6:**
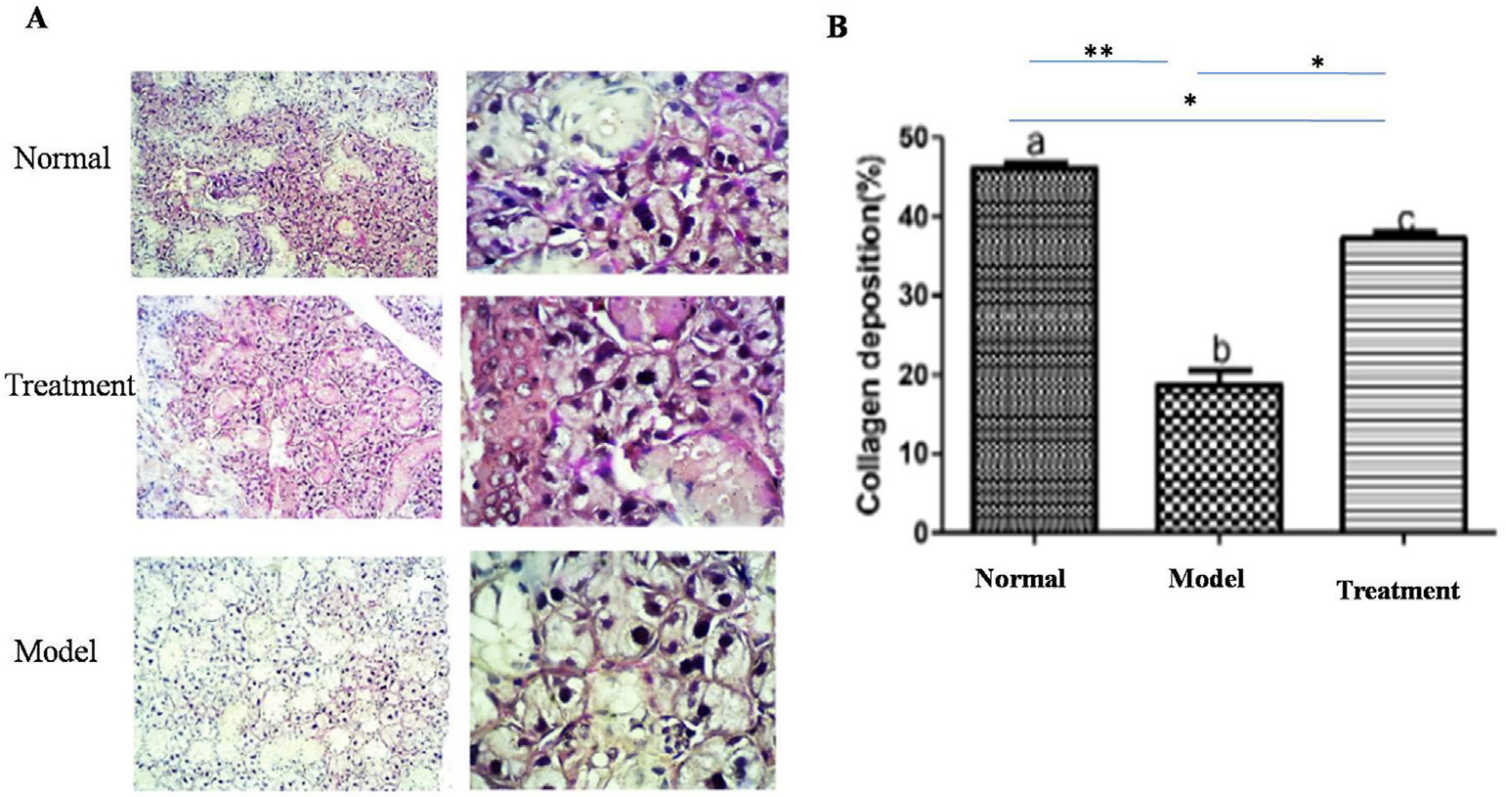
A: Alcian blue staining in various groups, B: The mean secretion of mucin in all group. ∗∗indicates P < 0.001, ∗ indicates P < 0.01.

PAS and Alcian blue staining indicated that the mean amounts of both serous and mucin secretions in the treatment group were increased and decreased compared to the positive and negative control groups, respectively (P < 0.001) (Figures 5 and 6).

### 3.4 Histopathological findings

**Figure 7:**
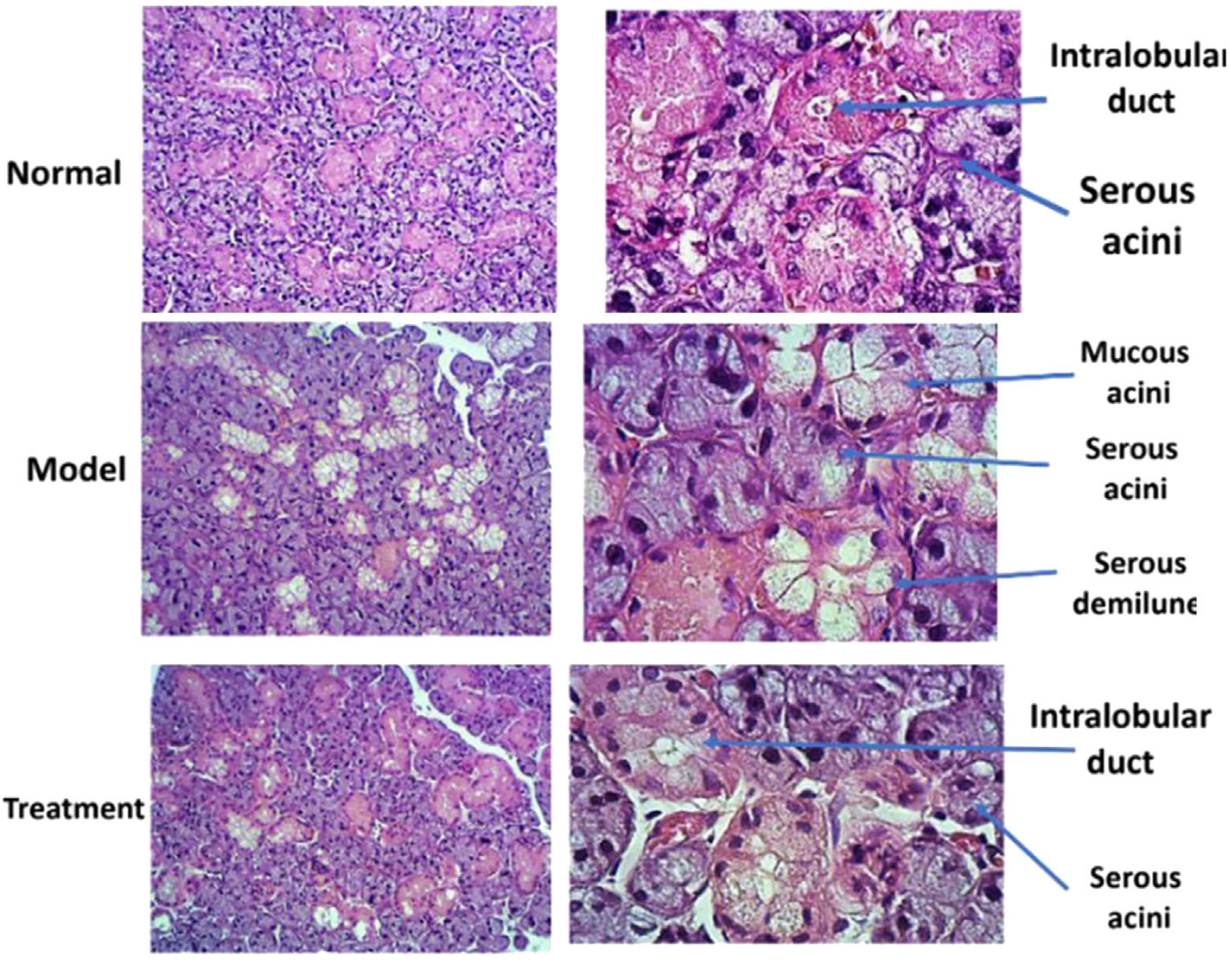
Histopathologic section of submandibular gland in various groups, H& E staining.

The histological sections were blindly examined by an expert pathologist for evaluation of the effect of cell therapy on SG structures. Hematoxylin and eosin staining showed that the overall structures of the submandibular gland tissue change morphologically. In the normal group, the density of serous acini was higher than that of mucous acini. However, in the treatment group, the density of serous acini was decreased compared to the normal group. In the positive control the density of both serous and mucous acini were considerably decreased compared to the normal or negative control group (Figure 7). Based on histological sections of the positive groups, the hitological changes were ranging from atrophic to necrotic. However, in the treatment group there was no necrotic area with focal atrophic area.

## 4 Discussion

The primary aim of this study was to investigate the potential ability of MSCs isolated from submandibular SG of healthy rats to restore the function and structure of the atrophic/necrotic SG in the rat animal model using intraoral duct ligation for one month. The atrophic effect of intraoral duct ligation, the main duct of the submandibular gland, has been previously reported (Osailan et al., 2006). The SGs are became atrophic and hypertrophic by reducing and increasing in size of the secretory cells, e.g., of acini and granular convoluted tubules, respectively (Hayashi et al., 2000). Temporary duct ligation alone led to reduction in SG size without the large scale necrosis. The necrotic SG is characterized with SG cells death associated with infiltration of inflammatory cells (Redman, 2008). Radiation-induced necrosis is irreversible injury and available treatments can only increase secretion from the remaining surviving tissue. MSC has been recently suggested to play a promising role as a regenerative agent against RAI-induced SG necrosis (Saylam et al., 2017). To detect the acinar cell protein secretory granules, Alcian Blue/PAS staining was done. Alcian Blue and PAS staining indicated that the mean amount of serous and mucin secretion in the treatment group was significantly increased compared to the positive control groups. These findings showed that the transplanted MSCs can regenerate the necrotic secretory duct and increase the acinar cell protein secretory granules. We have confirmed that the isolated and injected MSCs have regenerative and reparative effect on necrotic and atrophic SG functionally and structurally in a rat model on two weeks post-transplanted. Based on our knowledge this is the first study that investigate the potential effectiveness of MSCs in treatment of atrophic SGs in an animal model. Functionally and structurally, the epithelial tissues of salivary and lacrimal glands are destructed in the various diseases leading to dryness of the mouth (xerostomia). Xerostomia greatly impact the patient's quality of life (Porcheri and Mitsiadis, 2019). The available treatments for SG atrophy are temporary and limited to systemic parasympathomimetic drugs such as pilocarpine, sialogogues, and artificial saliva substitutes and SG is not regenerate functionally (See et al., 2019). New therapeutic options have shifted to regenerating SG cells by stem cell transplantation (Lombaert et al., 2017; Kim et al., 2019).

We have confirmed the nature of isolated stem cells using flow cytometery for CD105 and CD90 marker that are highly expressed in the MSCs. Cell-based therapy has been recently increasing attention for regeneration medicine such as damaged SGs (Lombaert et al., 2017; Kim et al., 2019). MSCs are alternative source of multipotent stem cells that have been widely used to differentiation of many types of cell lineages (Rashtbar et al., 2018; Shirian et al., 2016). They are the most suitable cells for regeneration of the most organs with self-renewal ability and their unique immunomodulatory and anti-inflammatory properties (Hoveizi et al., 2019) that were used as cell sources in this study. However, Xu et al. (2012) have shown a defective MSCs immunoregulatory function in Sjögren syndrome patients and NOD mice.

We have showed that locally injected MSCs regenerate the overall histological structures of the necrotic submandibular gland tissue on 2 weeks post-transplanted. On the other hand, despite the densities of serous and mucous acini in the treatment group were decreased compared to the normal group, both serous and mucous acini were considerably increased compared to the control group. The systemic administration of MSCs derived from adipose tissue has been reported to ameliorate RI-induced histologic changes and salivary dysfunction in a rat model (Kim et al., 2019).

For demonstration of mitotic index or proliferation rate of the SG epithelia tissue, Ki-67 and Smbg protein expression levels were evaluated using immunohistochemistry. The treatment group significantly expressed higher Ki-67 protein, as a diagnostic marker for cell mitosis and proliferation rate, and lower Smbg as a diagnostic marker for damage to the submandibular gland than that of the control group. However, the expression levels of Ki-67 and Smbg were significantly lower and higher in the treatment group than that of the normal group, respectively. These findings showed that the transplanted MSCs not only promote mitosis of the damaged SG but also may contribute to regenerate the atrophic and necrotic structures of SGs. These findings showed that transplanted MSCs may have exerted their regenerative effect either via differentiation of MSCs to SGs epithelial cells or promotion of stem cell of submandibular gland. The expression of Ki-67 as one of the several salivary and lacrimal glands markers involved in regeneration and proliferation has been recently demonstrated by Abughanam et al. (2019).

In conclusion, this study demonstrates the therapeutic benefits of MSCs isolated from SG in treating of atrophic and necrotic SG in a rat model. MSCs may be potential candidates for cell-based therapies in targeting hypofunction of SG induced by a verity of diseases or surgery as well as radiotherapy of head and neck cnacers.

## Declarations

### Author contribution statement

N. Bahrami: Conceived and designed the experiments.

S. Najafi, H. Nosrati, Z. Faraji, A. Mohamadnia, S.M. Mortazavi: Performed the experiments; Contributed reagents, materials, analysis tools or data.

S. Shirian: Analyzed and interpreted the data; Wrote the paper.

### Funding statement

This research did not receive any specific grant from funding agencies in the public, commercial, or not-for-profit sectors.

### Competing interest statement

The authors declare no conflict of interest.

### Additional information

No additional information is available for this paper.

## References

[bib1] Abughanam G., Elkashty O.A., Liu Y., Bakkar M.O., Tran S.D. (2019). Mesenchymal stem cells extract (MSCsE)-Based therapy alleviates xerostomia and keratoconjunctivitis sicca in sjogren’s syndrome-like disease. Int. J. Mol. Sci..

[bib2] Choi J.S., An H.Y., Shin H.S., Kim Y.M., Lim J.Y. (2018). Enhanced tissue remodelling efficacy of adipose-derived mesenchymal stem cells using injectable matrices in radiation-damaged salivary gland model. J. Tissue Eng. Regen. Med..

[bib3] de Paula F., Teshima T.H.N., Hsieh R., Souza M.M., Nico M.M.S., Lourenco S.V. (2017). Overview of human salivary glands: highlights of morphology and developing processes. Anat Rec. (Hoboken).

[bib4] Emmerson E., Knox S.M. (2018). Salivary gland stem cells: a review of development, regeneration and cancer. Genesis.

[bib5] Hayashi H., Ozono S., Watanabe K., Nagatsu I., Onozuka M. (2000). Morphological aspects of the postnatal development of submandibular glands in male rats: involvement of apoptosis. J. Histochem. Cytochem..

[bib6] Hoveizi E., Tavakol S., Shirian S., Sanamiri K. (2019). Electrospun nanofibers for diabetes: tissue engineering and cell-based therapies. Curr. Stem Cell Res. Ther..

[bib7] Kim J.W., Kim J.M., Choi M.E., Kim S.K., Kim Y.M., Choi J.S. (2019). Adipose-derived mesenchymal stem cells regenerate radioiodine-induced salivary gland damage in a murine model. Sci. Rep..

[bib8] Lim J.Y., Yi T., Choi J.S., Jang Y.H., Lee S., Kim H.J., Song S.U., Kim Y.M. (2013). Intraglandular transplantation of bone marrow-derived clonal mesenchymal stem cells for amelioration of post-irradiation salivary gland damage. Oral Oncol..

[bib9] Lombaert I.M., Brunsting J.F., Wierenga P.K. (2008). Rescue of salivary gland function after stem cell transplantation in irradiated glands. PloS One.

[bib10] Lombaert I., Movahednia M.M., Adine C., Ferreir J.N. (2017). Concise review: salivary gland regeneration: therapeutic approaches from stem cells to tissue organoids. Stem Cell..

[bib11] Mattingly A.F., Finley J.K., Knox S.M. (2015). Salivary gland development and disease. Wiley Interdiscip. Rev. Dev. Biol..

[bib12] Millsop J.W., Wang E.A., Fazel N. (2017). Etiology, evaluation, and management of xerostomia. Clin. Dermatol..

[bib13] Nanduri L.S., Maimets M., Pringle S.A., van der Zwaag M., van Os R.P., Coppes R.P. (2011). Regeneration of irradiated salivary glands with stem cell marker expressing cells. Radiother. Oncol..

[bib14] Nicolay N.H., Lopez Perez R., Debus J., Huber P.E. (2015). Mesenchymal stem cells - a new hope for radiotherapy-induced tissue damage?. Canc. Lett..

[bib15] Osailan S.M., Proctor G.B., McGurk M., Paterson K.L. (2006). Intraoral duct ligation without inclusion of the parasympathetic nerve supply induces rat submandibular gland atrophyInt. J. Exp. Pathol..

[bib16] Porcheri C., Mitsiadis T.A. (2019). Physiology, pathology and regeneration of salivary glands. Cells.

[bib17] Rashtbar M., Hadjati J., Ai J., Shirian S., Jahanzad I., Azami M., Asadpuor S., Sadroddiny E. (2018). Critical-sized full-thickness skin defect regeneration using ovine small intestinal submucosa with or without mesenchymal stem cells in rat model. J. Biomed. Mater. Res. B Appl. Biomater..

[bib18] Redman R.S. (2008). On approaches to the functional restoration of salivary glands damaged by radiation therapy for head and neck cancer, with a review of related aspects of salivary gland morphology and development. Biotech. Histochem..

[bib19] Saylam G., Ö Bayır, Gültekin S.S., Pınarlı F.A., Han Ü., Korkmaz M.H., Sancaktar M.E., Tatar İ., Sargon M.F., Tatar E.Ç. (2017). Protective/restorative role of the adipose tissue-derived mesenchymal stem cells on the radioiodine-induced salivary gland damage in rats. Radiol. Oncol..

[bib20] See L., Mohammadi M., Han P.P., Mulligan R., Enciso R. (2019). Efficacy of saliva substitutes and stimulants in the treatment of dry mouth. Spec. Care Dent..

[bib21] Shirian S., Ebrahimi-Barough S., Saberi H., Norouzi-Javidan A., Mousavi S.M., Derakhshan M.A., Arjmand B., Ai J. (2016). Comparison of capability of human bone marrow mesenchymal stem cells and endometrial stem cells to differentiate into motor neurons on electrospun poly(ε-caprolactone) scaffold. Mol. Neurobiol..

[bib22] Soleimanpuor H., Shirian S., Oryan A., Daneshbod K., Bagheri N., Daneshbod Y. (2012). Cytological, immunocytochemical, histological, and immunohistochemical diagnosis of poorly differentiated Sertoli-Leydig cell tumor. Acta Cytol..

[bib23] Sui Y., Zhang S., Li Y., Zhang X., Hu W., Feng Y., Xiong J., Zhang Y., Wei S. (2020). Generation of functional salivary gland tissue from human submandibular gland stem/progenitor cells. Stem Cell Res. Ther..

[bib24] Xu J., Wang D., Liu D., Fan Z., Zhang H., Liu O., Ding G., Gao R., Zhang C., Ding Y., Bromberg J.S., Chen W., Sun L., Wang S. (2012). Allogeneic mesenchymal stem cell treatment alleviates experimental and clinical Sjögren syndrome. Blood.

